# Association of Chronic Kidney Disease and Peripheral Artery Disease with Inappropriate Left Ventricular Mass

**DOI:** 10.1371/journal.pone.0048422

**Published:** 2012-10-31

**Authors:** Ho-Ming Su, Tsung-Hsien Lin, Po-Chao Hsu, Chee-Siong Lee, Wen-Hsien Lee, Szu-Chia Chen, Wen-Chol Voon, Wen-Ter Lai, Sheng-Hsiung Sheu

**Affiliations:** 1 Division of Cardiology, Department of Internal Medicine, Kaohsiung Medical University Hospital, Kaohsiung Medical University, Kaohsiung, Taiwan; 2 Division of Nephrology, Department of Internal Medicine, Kaohsiung Medical University Hospital, Kaohsiung Medical University, Kaohsiung, Taiwan; 3 Department of Internal Medicine, Kaohsiung Municipal Hsiao-Kang Hospital, Kaohsiung Medical University, Kaohsiung, Taiwan; 4 Faculty of Medicine, College of Medicine, Kaohsiung Medical University, Kaohsiung, Taiwan; INRCA, Italy

## Abstract

Inappropriate left ventricular mass index (LVM) may develop as a response to particular hemodynamic and metabolic alterations. Inappropriate LVM and peripheral artery disease (PAD) characterized by abnormally low or high ankle-brachial index (ABI) are common in chronic kidney disease (CKD) patients, in whom there may be a close and cause-effect relationship. The aim of this study is to assess whether CKD and abnormal ABI has an independent and additive association with inappropriate LVM. A total of 1110 patients were included in the study. Inappropriate LVM was defined as observed LVM more than 28% of the predicted value. The ABI was measured using an ABI-form device. PAD was defined as ABI <0.9 or >1.3 in either leg. Multivariate analysis showed that patients with estimated glomerular filtration rate (eGFR) <45 ml/min/1.73 m^2^ (odds ratio [OR], 1.644; *P* = 0.011) and PAD (OR, 2.082; *P* = 0.002) were independently associated with inappropriate LVM. The interaction between eGFR <45 ml/min/1.73 m^2^ and PAD on inappropriate LVM was statistically significant (*P* = 0.044). Besides, eGFR<45 ml/min/1.73 m^2^ (change in observed/predicted LVM, 19.949; *P*<0.001) and PAD (change in observed/predicted LVM, 11.818; *P* = 0.003) were also significantly associated with observed/predicted LVM. Our findings show that eGFR <45 ml/min/1.73 m^2^ and PAD are independently and additively associated with inappropriate LVM and observed/predicted LVM. Assessments of eGFR and ABI may be useful in identifying patients with inappropriate LVM.

## Introduction

A condition of growth of myocardium exceeding the hemodynamic needs has been reported and called inappropriate left ventricular mass (LVM). Predicted LVM for sex, height^2.7^, and hemodynamic load can be used as an inappropriate reference for the observed LVM [1], [2]. Recently, the presence of inappropriate LVM has been found in a significant proportion of patients with arterial hypertension, aortic stenosis, or chronic kidney disease (CKD) and has been reported to have a negative impact on cardiovascular prognosis [1], [3]–[7]. Therefore, identifying patients with inappropriate LVM for aggressive treatment interventions is important.

Left ventricular hypertrophy (LVH) is not only highly prevalent in CKD patients but also often inappropriate [1], [6], [7]. Besides, high prevalence of peripheral artery disease (PAD) characterized by abnormal ankle-brachial index (ABI) is frequently noted in patients with CKD [8], [9]. Abnormally low and high ABIs are affected by or linked to various risk factors for LVH, such as arterial stiffness, hypertension, and coexisting atherosclerosis [10], [11]. There may be a close and cause-effect relationship between CKD, PAD and inappropriate LVM. However, there are limited studies to evaluate the association of CKD and PAD with inappropriate LVM. Accordingly, the aim of this study is to assess whether CKD and abnormal ABI has an independent and additive association with inappropriate LVM.

## Subjects and Methods

### Study Patients and Design

Study subjects were randomly included from a group of patients who arranged for echocardiographic examinations at Kaohsiung Municipal Hsiao-Kang Hospital. Patients with significant aortic or mitral valve disease, atrial fibrillation, or inadequate image visualization were excluded. We did not include all patients consecutively because ABI and blood pressures must be measured within 5 min after the completion of an echocardiographic examination. A total of 1110 patients (mean age 61.3±13.8 years, 646 males/464 females) were included.

### Ethics Statement

The study protocol was approved by the institutional review board of the Kaohsiung Medical University Hospital (KMUH-IRB-20120131). Informed consents have been obtained in written form from patients and all clinical investigation was conducted according to the principles expressed in the Declaration of Helsinki. The patients gave consent for the publication of the clinical details.

### Evaluation of Cardiac Structure and Function

The echocardiographic examination was performed by one experienced cardiologist with a VIVID 7 (General Electric Medical Systems, Horten, Norway), with the participant respiring quietly in the left decubitus position. The cardiologist was blind to the other data. Two-dimensional and two-dimensionally guided M-mode images were recorded from the standardized views. The echocardiographic measurements included left ventricular internal diameter in diastole (LVIDd), left ventricular posterior wall thickness in diastole (LVPWTd), interventricular septal wall thickness in diastole (IVSTd), E-wave deceleration time, transmitral E wave velocity and transmitral A wave velocity. Left ventricular ejection fraction (LVEF) was measured by the modified Simpson’s method. Left ventricular relative wall thickness (LVRWT) was calculated as the ratio of 2×LVPWTd/LVIDd. Observed LVM was calculated using Devereux-modified method, i.e. LVM = 1.04× [(IVSTd + LVIDd + LVPWTd)^3^– LVIDd^3^] –13.6 g [12]. Predicted LVM was estimated using the following equation [2]: predicted LVM = 55.37+6.64×height (m^2.7^) +0.64×stroke work - 18.07×sex (in which sex was coded as male = 1 and female = 2). Stroke work was estimated as systolic blood pressure times stroke volume product and converted in gram meters by multiplying by 0.0144. Inappropriate LVM was also assessed as the ratio between observed and predicted LVM (observed/predicted LVM). LVM was defined ‘inappropriate’ when observed LVM was more than 28% of the predicted value (i.e. observed/predicted LVM >128%) [1], [2].

### Assessment of ABI

The values of ABI were measured by using an ABI-form device (VP1000; Colin Co. Ltd., Komaki, Japan), which automatically and simultaneously measured blood pressures in both arms and ankles using an oscillometric method [13]–[15]. The ABI was calculated by the ratio of the ankle systolic blood pressure divided by the arm systolic blood pressure. The ABI measurement was done once in each patient. PAD was defined as ABI <0.9 or ≥1.3 in either leg.

### Collection of Demographic, Medical and Laboratory Data

Demographic and medical data, including age, gender and comorbid conditions were garnered from medical records or interviews with patients. Body mass index (BMI) was calculated as the ratio of weight in kilograms divided by square of height in meters. Blood samples were obtained within 1 month of enrollment. Laboratory data were measured from fasting blood samples using an autoanalyzer (Roche Diagnostics GmbH, D-68298 Mannheim COBAS Integra 400). Serum creatinine was measured by the compensated Jaffé (kinetic alkaline picrate) method in a Roche/Integra 400 Analyzer (Roche Diagnostics, Mannheim, Germany) using a calibrator traceable to isotope-dilution mass spectrometry [16]. The value of estimated glomerular filtration rate (eGFR) was calculated using the 4-variable equation in the Modification of Diet in Renal Disease (MDRD) study [17]. In addition, information regarding antihypertensive medications including angiotensin converting enzyme inhibitors (ACEI), angiotensin II receptor blockers (ARB), β-blockers, calcium channel blockers and diuretics during the study period was obtained from medical records.

### Statistical Analysis

Statistical analysis was performed using SPSS 15.0 for windows (SPSS Inc. Chicago, USA). Data are expressed as percentages, mean ± standard deviation or median (25^th^–75^th^ percentile) for triglyceride. Multiple comparisons among the study groups were performed by one-way analysis of variance (ANOVA) followed by post hoc test adjusted with a Boneferroni correction. The differences between patients with appropriate and inappropriate LVM were checked by Chi-square test for categorical variables or by independent t-test for continuous variables. Age, sex and non-echocardiographic variables which were significantly different between patients with appropriate and inappropriate LVM were selected for multivariate analysis. Hence, the adjusted covariates included age, sex, diabetes mellitus (DM), hypertension, coronary artery disease, PAD, mean arterial pressure, pulse pressure, BMI, log triglyceride, eGFR <45 ml/min/1.73 m^2^, 4 study groups and medication including ACEI and/or ARB, β-blocker and diuretic use. Multiple logistic and linear regression analyses were used to identify the factors associated with inappropriate LVM and observed/predicted LVM. A difference was considered significant if the *P* value was less than 0.05.

## Results

As can be seen in Table 1, the comparison of baseline and echocardiographic characteristics between patients with appropriate and inappropriate LVM, we studied 1110 patients (646 men and 464 women, mean age 61.3±13.8 years). The prevalence of inappropriate LVM was 68.3% and the value of observed/predicted LVM of all patients was 153.2±48.3%. Compared with patients with appropriate LVM, patients with inappropriate LVM were significantly associated with more male, higher prevalence of DM, higher prevalence of hypertension, higher prevalence of coronary artery disease, higher prevalence of PAD, lower mean arterial pressure, lower pulse pressure, higher BMI, higher triglyceride, lower eGFR, higher prevalence of eGFR <45 mL/min/1.73 m^2^, more ACEI and/or ARB use, more β-blocker use, more diuretic use, lower LVEF and higher LVRWT. The prevalence of eGFR <45 mL/min/1.73 m^2^ was higher in patients with inappropriate LVM (19.3% and 25.7%, *P* = 0.019), but the prevalence of eGFR <60 mL/min/1.73 m^2^ was comparable between the two groups (47.4% and 52.6%, *P* = 0.107).

**Table 1:** Comparison of baseline and echocardiographic characteristics between patients with appropriate and inappropriate left ventricular mass.

Characteristics	All patients (n = 1110)	Appropriate LVM (n = 352)	Inappropriate LVM (n = 758)
Age (year)	61.3±13.8	60.9±14.3	61.4±13.5
Male gender (%)	58.2	52.3	60.9*
Diabetes mellitus (%)	29.1	23.9	31.5*
Hypertension (%)	71.1	66.5	73.7*
Coronary artery disease (%)	19.8	15.3	21.9*
PAD (%)	15.7	9.4	18.6**
Mean arterial pressure (mmHg)	97.2±14.0	99.7±13.0	96.0±14.3**
Pulse pressure (mmHg)	58.6±14.6	61.2±14.3	57.4±14.5**
Body mass index (kg/m^2^)	26.1±4.0	24.8±3.5	26.7±4.1**
Laboratory parameters			
Albumin (g/dL)	4.07±0.45	4.12±0.43	4.05±0.46
Fasting glucose (mg/dL)	114.2±41.5	112.7±41.2	115.0±41.6
Triglyceride (mg/dL)	124 (86–186)	118 (83–171)	128 (87–190.75)*
Total cholesterol (mg/dL)	191.2±43.8	193.1±42.8	190.3±44.2
Hematocrit (%)	40.2±5.6	40.2±5.0	40.1±5.9
Baseline eGFR (mL/min/1.73 m^2^)	57.0±20.7	59.2±19.7	56.0±21.1*
eGFR <45 mL/min/1.73 m^2^ (%)	23.7	19.3	25.7*
eGFR <60 mL/min/1.73 m^2^ (%)	51.0	47.4	52.6
Medications			
ACEI and/or ARB use (%)	56.8	46.1	61.5**
β-blocker use (%)	43.5	38.0	46.0*
Calcium channel blocker use (%)	36.8	33.0	38.6
Diuretic use (%)	28.8	16.5	34.5**
Echocardiographic data			
Observed/predicted LVM (%)	153.2±48.3	108.7±16.6	173.8±44.0**
LVEF (%)	60.9±14.5	65.6±10.2	58.7±15.6**
LVRWT	0.38±0.09	0.35±0.07	0.40±0.09**
E-wave deceleration time (ms)	204.8±67.1	201.4±60.0	206.4±70.2
E/A	1.01±0.52	1.01±0.47	1.00±0.55

Abbreviations. LVM, left ventricular mass; PAD, peripheral artery disease; eGFR, estimated glomerular filtration rate; ACEI, angiotensin converting enzyme inhibitor; ARB, angiotensin II receptor blocker; LVEF, left ventricular ejection fraction; LVRWT, left ventricular relative wall thickness; E, transmitral E wave velocity; A, transmitral A wave velocity.

* *P*<0.05, **P<0.001 compared to patients with appropriate LVM.

The study population was further classified into four groups according to eGFR ≥45 or <45 ml/min/1.73 m^2^ and with/without PAD. Groups 1, 2, 3 and 4 were made up of patients with eGFR ≥45 ml/min/1.73 m^2^ without PAD, eGFR <45 ml/min/1.73 m^2^ without PAD, eGFR ≥45 ml/min/1.73 m^2^ with PAD and eGFR <45 ml/min/1.73 m^2^ with PAD, respectively. The comparison of clinical characteristics among the study groups was shown in Table 2. There were 737, 199, 110 and 644 patients in groups 1, 2, 3 and 4, respectively. The observed/predicted LVM in groups 1, 2, 3 and 4 were 147.3±43.6, 162.1±58.3, 159.2±46.5 and 182.7±52.8%, respectively. Additionally, the prevalence of inappropriate LVM in groups 1, 2, 3 and 4 were 64.7%, 70.4%, 78.2% and 87.5%, respectively.

**Table 2:** Comparison of baseline and echocardiographic characteristics among 4 study groups.

Characteristics	*Group 1* (n = 737)	*Group 2* (n = 199)	*Group 3* (n = 110)	*Group 4* (n = 64)
Age (year)	59.0±13.2	65.4±13.1*	63.4±15.1*	70.3±12.5* #
Male gender (%)	57.3	55.3	72.7* †	53.1
Diabetes mellitus (%)	22.8	40.2*	27.3	70.3* † ^#^
Hypertension (%)	66.5	78.9	76.1	92.2*
Coronary artery disease (%)	17.6	19.2	38.2* †	15.6#
Mean arterial pressure (mmHg)	96.5±13.2	100.4±16.3*	95.1±13.9†	98.2±14.2
Pulse pressure (mmHg)	56.1±13.2	64.1±16.3*	59.4±15.1†	68.8±14.3* #
Body mass index (kg/m^2^)	26.2±3.9	25.4±3.8	26.3±4.1	26.5±5.3
Laboratory parameters				
Albumin (g/dL)	4.18±0.37	3.90±0.50*	4.07±0.53	3.79±0.49* #
Fasting glucose (mg/dL)	111.7±37.7	119.0±50.4	116.0±46.1	128.8±43.3*
Triglyceride (mg/dL)	122.5 (84.75–183)	125.5 (90–189.75)	138 (84.5–210.5)	116.5 (87.25–169)
Total cholesterol (mg/dL)	194.9±44.1	180.4±42.5*	188.0±43.0	186.9±39.6
Hematocrit (%)	42.0±4.3	35.8±6.1*	40.5±5.7* †	34.1±5.5* #
Baseline eGFR(mL/min/1.73 m^2^)	66.3±13.3	29.0±12.7*	63.4±12.5†	25.9±12.3* #
Medications				
ACEI and/or ARB use (%)	54.1	61.8	57.3	71.9*
β–blocker use (%)	42.4	48.7	45.5	35.9
Calcium channel blocker use (%)	31.0	48.2*	40.9	60.9* #
Diuretic use (%)	24.7	36.4*	26.4	56.3* † ^#^
Echocardiographic data				
Observed/predicted LVM (%)	147.3±43.6	162.1±58.3*	159.2±46.5	182.7±52.8* † ^#^
Inappropriate LVM (%)	64.7	70.4	78.2*	87.5*
LVEF (%)	62.3±13.6	58.8±16.2*	59.2±15.1	60.0±15.6
Relative wall thickness	0.38±0.09	0.38±0.09	0.37±0.10	0.41±0.10#
E-wave deceleration time (ms)	198.0±59.9	215.9±79.9*	224.1±76.8*	216.1±74.6
E/A	1.02±0.53	0.94±0.51	0.97±0.45	1.06±0.56

Groups 1, 2, 3 and 4 were made up of patients with eGFR ≥45 ml/min/1.73 m^2^ without PAD, eGFR <45 ml/min/1.73 m^2^ without PAD, eGFR ≥45 ml/min/1.73 m^2^ with PAD and eGFR <45 ml/min/1.73 m^2^ with PAD, respectively. Abbreviations are the same as in Table 1.

* *P*<0.05 compared with group 1;

† *P*<0.05 compared with group 2;

# *P*<0.05 compared with group 3.

### Relation of eGFR <45 ml/min/1.73 m^2^ and PAD to Inappropriate LVM and Observed/predicted LVM

Table 3 shows the odds ratio (OR) estimates for inappropriate LVM and the unstandardized coefficient β estimates for observed/predicted LVM with adjustment for age, sex, DM, hypertension, coronary artery disease, PAD, mean arterial pressure, pulse pressure, BMI, log triglyceride, eGFR <45 mL/min/1.73 m^2^ and medication including ACEI and/or ARB, β-blocker and diuretic use. In the multivariate logistic analysis, eGFR <45 mL/min/1.73 m^2^ (OR, 1.644; 95% confidence interval [CI], 1.120 to 2.414; *P* = 0.011) and PAD (OR, 2.082; 95% CI, 1.311 to 3.307; *P* = 0.002) were significantly associated with inappropriate LVM. The interaction between eGFR <45 ml/min/1.73 m^2^ and PAD on inappropriate LVM was statistically significant (OR, 2.982; 95% CI, 1.456 to 6.104; *P* = 0.003). As for the relation of eGFR <45 mL/min/1.73 m^2^ and PAD to observed/predicted LVM, the multivariate linear analysis shows eGFR <45 mL/min/1.73 m^2^ (change in observed/predicted LVM, 19.949; 95% CI 13.098 to 26.800; *P*<0.001) and PAD (change in observed/predicted LVM, 11.818; 95% CI, 4.144 to 19.493; *P* = 0.003) were independently associated with observed/predicted LVM.

**Table 3:** Multivariate analyses of eGFR <45 mL/min/1.73 m^2^ and PAD to inappropriate LVM and observed/predicted LVM.

Parameters	inappropriate LVM		observed/predicted LVM	
	Odds ratio (95% CI)	*P*	Unstandardized coefficient β (95% CI)	*P*
eGFR <45 mL/min/1.73 m^2^	1.644 (1.120–2.414)	0.011	19.949 (13.098–26.800)	<0.001
PAD	2.082 (1.311–3.307)	0.002	11.818 (4.144–19.493)	0.003

Multivariate model: adjusted for age, sex, diabetes mellitus, hypertension, coronary artery disease, PAD, mean arterial pressure, pulse pressure, body mass index, log triglyceride, eGFR <45 mL/min/1.73 m^2^ and medication including ACEI and/or ARB, β-blocker and diuretic use. Abbreviations are the same as in Table 1.

### Relation of Study Groups to Inappropriate LVM and Observed/predicted LVM

Table 4 displays the OR estimates for inappropriate LVM and the unstandardized coefficient β estimates for observed/predicted LVM with adjustment for age, sex, DM, hypertension, coronary artery disease, 4 study groups, mean arterial pressure, pulse pressure, BMI, log triglyceride, and medication including ACEI and/or ARB, β-blocker and diuretic use. In the multivariate logistic analysis, group 2 (OR, 1.626; 95% CI, 1.078 to 2.453; *P* = 0.020; *vs.* group 1), group 3 (OR, 2.042; 95% CI, 1.201 to 3.471; *P* = 0.008; *vs.* group 1) and group 4 (OR, 3.592; 95% CI, 1.481 to 8.712; *P* = 0.005; *vs.* group 1) were significantly associated with inappropriate LVM. As for the relation of study groups to observed/predicted LVM, the multivariate linear analysis shows group 2 (change in observed/predicted LVM, 20.293; 95% CI 12.725 to 27.862; *P*<0.001; *vs.* group 1), group 3 (change in observed/predicted LVM, 12.356; 95% CI, 3.186 to 21.526; *P* = 0.008; *vs.* group 1) and group 4 (change in observed/predicted LVM, 30.865; 95% CI, 17.796 to 43.934; *P*<0.001; *vs.* group 1) were independently associated with observed/predicted LVM.

**Table 4:** Multivariate analyses of study groups to inappropriate LVM and observed/predicted LVM.

Parameters	inappropriate LVM		observed/predicted LVM	
	Odds ratio (95% CI)	*P*	Unstandardized coefficient β (95% CI)	*P*
*Group 1*	1		1	
*Group 2*	1.626 (1.078–2.453)	0.020	20.293 (12.725–27.862)	<0.001
*Group 3*	2.042 (1.201–3.471)	0.008	12.356 (3.186–21.526)	0.008
*Group 4*	3.592 (1.481–8.712)	0.005	30.865 (17.796–43.934)	<0.001

Multivariate model: adjusted for age, sex, diabetes mellitus, hypertension, coronary artery disease, 4 study groups, mean arterial pressure, pulse pressure, body mass index, log triglyceride and medication including ACEI and/or ARB, β-blocker and diuretic use. Groups 1, 2, 3 and 4 were made up of patients with eGFR ≥45 ml/min/1.73 m^2^ without PAD, eGFR <45 ml/min/1.73 m^2^ without PAD, eGFR ≥45 ml/min/1.73 m^2^ with PAD and eGFR <45 ml/min/1.73 m^2^ with PAD, respectively. Abbreviations are the same as in Table 1.

## Discussion

In the present study, we evaluated the association of CKD and PAD with inappropriate LVM and observed/predicted LVM and found eGFR <45 ml/min/1.73 m^2^ and PAD were independently and additively associated with inappropriate LVM and observed/predicted LVM. In addition, patients with coexistence of eGFR <45 ml/min/1.73 m^2^ and PAD had the highest value of observed/predicted LVM among 4 study groups.

The ABI is reported to be a good marker for atherosclerosis and an ABI <0.9 is useful in the diagnosis of peripheral artery occlusive disease (PAOD) [18]–[20]. In addition, an ABI ≥1.3 is considered to indicate medial artery calcification (MAC) [21]. High prevalence of PAOD and increased MAC are frequently noted in patients with CKD [8], [9], which may through multiple pathogenic mechanisms involved, including deranged calcium/phosphate balance, secondary hyperparathyroidism, homocysteine, lipoprotein(a) metabolism, alterations in inflammatory and coagulation pathways, fluid overload, alterations in the angiotensin and endothelin systems, malnutrition, uremic toxins, oxidative stress and insulin resistance [22]–[26]. Moreover, either abnormally low or high ABI can predict overall and cardiovascular mortality in patients with chronic renal failure [27], [28]. In our study, eGFR <45 ml/min/1.73 m^2^ and PAD were independently and additively associated with inappropriate LVM and observed/predicted LVM even after adjustment for many confounding factors. Hence, it suggested that CKD and PAD might have a synergic effect on inappropriate LVM.

Another finding of our study was that when compared between the two groups without PAD, only the group with eGFR <45 ml/min/1.73 m^2^ was associated with inappropriate LVM. Recently, we also consistently demonstrated a significant trend for a stepwise increase in the observed/predicted LVM and in the prevalence of inappropriate LVM corresponding to advancement in CKD stages in CKD patients. Moreover, increased observed/predicted LVM was significantly associated with increased cardiovascular events in this population [7]. The possible mechanisms is that the hemodynamic and metabolic disturbances in patients with CKD may synergistically activate a variety of pathophysiological alterations including hemodynamic abnormalities (i.e. increased preload and afterload) and non-hemodynamic abnormalities (i.e. neuro-hormonal stressors, factors promoting myocardial fibrosis and atherosclerosis) and thus result in the excessive growth of LVM [29]–[33].

Similarly, when compared between the two groups without CKD, only the group with PAD was associated with inappropriate LVM. Previous studies demonstrated that the ABI value in the LVH group was significantly lower than that in the non-LVH group [34], [35]. In addition, Ix JH et al. [36] evaluated the association between abnormally low or high ABI and LVMI in 4972 MESA (multi-Ethic Study of atherosclerosis) participants without clinical cardiovascular disease. They found either abnormally low or high ABI was significantly associated with greater left ventricular mass index. Our recent study in CKD patients also showed patients with abnormal ABIs had a greater LVMI [37]. These results suggested that low and high ABI might be related to LVH. Atherosclerosis directly caused a decrease in blood perfusion in the lower extremities and an increase in arterial wall stiffness, contributing to arterial distensibility, and then final progressed to LVH [9], [38], [39].

The definition and classification for CKD was proposed by the National Kidney Disease National Kidney Foundation Kidney Disease Outcomes Quality Initiative (NKF-KDOQI) in 2002 [40]. CKD was defined based on the presence of kidney damage or eGFR <60 ml/min/1.73 m^2^ for more than 3 months, irrespective of cause. The high prevalence of early CKD stages may represent over-diagnosis of kidney disease and lead to the potential for overuse of substantially resources. Some have proposed a lower eGFR threshold (30 or 45 *vs.* 60 ml/min/1.73 m^2^) to define the presence of CKD or to subdivide stage 3 into stage 3a (45–59 ml/min/1.73 m^2^) and 3b (30–44 ml/min/1.73 m^2^) [41], [42]. In our study, eGFR <45 ml/min/1.73 m^2^ was significantly associated with inappropriate LVM (*P* = 0.019), but eGFR <60 ml/min/1.73 m^2^ did not achieve significance (*P* = 0.107). This finding supported the suggestion to lower the eGFR threshold for defining CKD. However, our study was only a cross-sectional one and lacked prognosis data. Thus, longer follow-up is required to clarify whether eGFR <45 ml/min/1.73 m^2^ is a better prognostic parameter than eGFR <60 ml/min/1.73 m^2^.

De Simone et al. [6] had evaluated the relations between inappropriate LVM and renal function in patients with CKD without known cardiac disease and found the prevalence of inappropriate LVM was higher in CKD patients than in healthy controls (43% *vs.* 10%, *P*<0.001). Besides, prevalence of inappropriate LVM paralleled the severity of renal dysfunction ranging from 10% in stage 1 CKD patients to 100% in stage 5 CKD patients. Nardi et al. [1] also investigated the comparison of the prevalence of inappropriate LVM between hypertensive patients with or without CKD. They found that the prevalence of inappropriate LVM was 52.5% in patients with CKD vs. 30.5% in hypertensive patients with normal renal function (*P*<0.001). In our study, the prevalence of inappropriate LVM was up to 68.3%. Because our study subjects were included from patients who arranged for echocardiographic examinations, they frequently had multiple comorbidities, such as DM, hypertension, coronary artery disease, PAD and CKD, which might partially explain the high prevalence of inappropriate LVM in our study.

There were several limitations to our study. Predicted LVM is calculated by age, gender, height and stroke work. Thus, a single blood pressure measurement may have a great impact on the calculation of predicted LVM. Average ambulatory blood pressure over 24 h may be more closely related to LVM than single clinic blood pressure measurement. In addition, treatment with antihypertensive drugs can potentially influence left ventricular geometry and functional parameters. In particular, the use of diuretics may reduce left ventricular diameter and thus cause a greater prevalence of inappropriate LVM. For ethical reason, we didn’t hold any drugs at the time of echocardiographic evaluation. However, in order to elucidate the influence of drugs, we had added different classes of antihypertensive drugs in the analysis and still found CKD and PAD had a significant association with inappropriate LVM and observed/predicted LVM.

In conclusion, our study demonstrated that eGFR <45 ml/min/1.73 m^2^ and PAD were independently and additively associated with inappropriate LVM and observed/predicted LVM. The combination of CKD and PAD might have a synergic effect on inappropriate LVM. Assessments of eGFR and ABI may be useful in identifying patients with inappropriate LVM.
